# Author Correction: Genomic analyses provide insights into spinach domestication and the genetic basis of agronomic traits

**DOI:** 10.1038/s41467-022-28320-w

**Published:** 2022-01-28

**Authors:** Xiaofeng Cai, Xuepeng Sun, Chenxi Xu, Honghe Sun, Xiaoli Wang, Chenhui Ge, Zhonghua Zhang, Quanxi Wang, Zhangjun Fei, Chen Jiao, Quanhua Wang

**Affiliations:** 1 Shanghai Engineering Research Center of Plant Germplasm Resources, College of Life Sciences, Shanghai Normal University, 200234 Shanghai, China; 2 Qinghai Key Laboratory of Vegetable Genetics and Physiology, Qinghai University, 810016 Xining, China; 3 Boyce Thompson Institute, Cornell University, Ithaca, NY 14853 USA; 4 College of Agriculture and Food Science, Zhejiang A&F University, 311300 Hangzhou, China; 5 Plant Biology Section, School of Integrative Plant Science, Cornell University, Ithaca, NY 14853 USA; 6 Engineering Laboratory of Genetic Improvement of Horticultural Crops of Shandong Province, College of Horticulture, Qingdao Agricultural University, 266109 Qingdao, China; 7 College of Life Science and Technology, Harbin Normal University, 150025 Harbin, China; 8 USDA-ARS, Robert W. Holley Center for Agriculture and Health, Ithaca, NY 14853, 18 USA; 9 Key Lab of Molecular Biology of Crop Pathogens and Insects, Institute of Biotechnology, Zhejiang University, 310058 Hangzhou, China

**Keywords:** Plant domestication, Plant genetics, Agricultural genetics, Genomics

Correction to: *Nature Communications* 10.1038/s41467-021-27432-z, published online 13 December 2021.

In the original version of this Article, Taiwan was coloured differently from mainland China in the map presented in Figure 3a. The map has now been updated according to authors’ request in both PDF and HTML version of the Article. Springer Nature remains neutral with regard to jurisdictional claims in published maps and institutional affiliations.

