# Supplementary material for: Genomic analyses provide insights into spinach domestication and the genetic basis of agronomic traits
Source: Nat Commun. 2021 Dec 13;12:7246. doi: 10.1038/s41467-021-27432-z (PMC8668906; doi:10.1038/s41467-021-27432-z)
Supplement: Supplementary file 1 — Supplementary Information [file 41467_2021_27432_MOESM1_ESM.pdf]

Genomic analyses provide insights into spinach domestication and the genetic basis of agronomic traits

Cai *et al.*

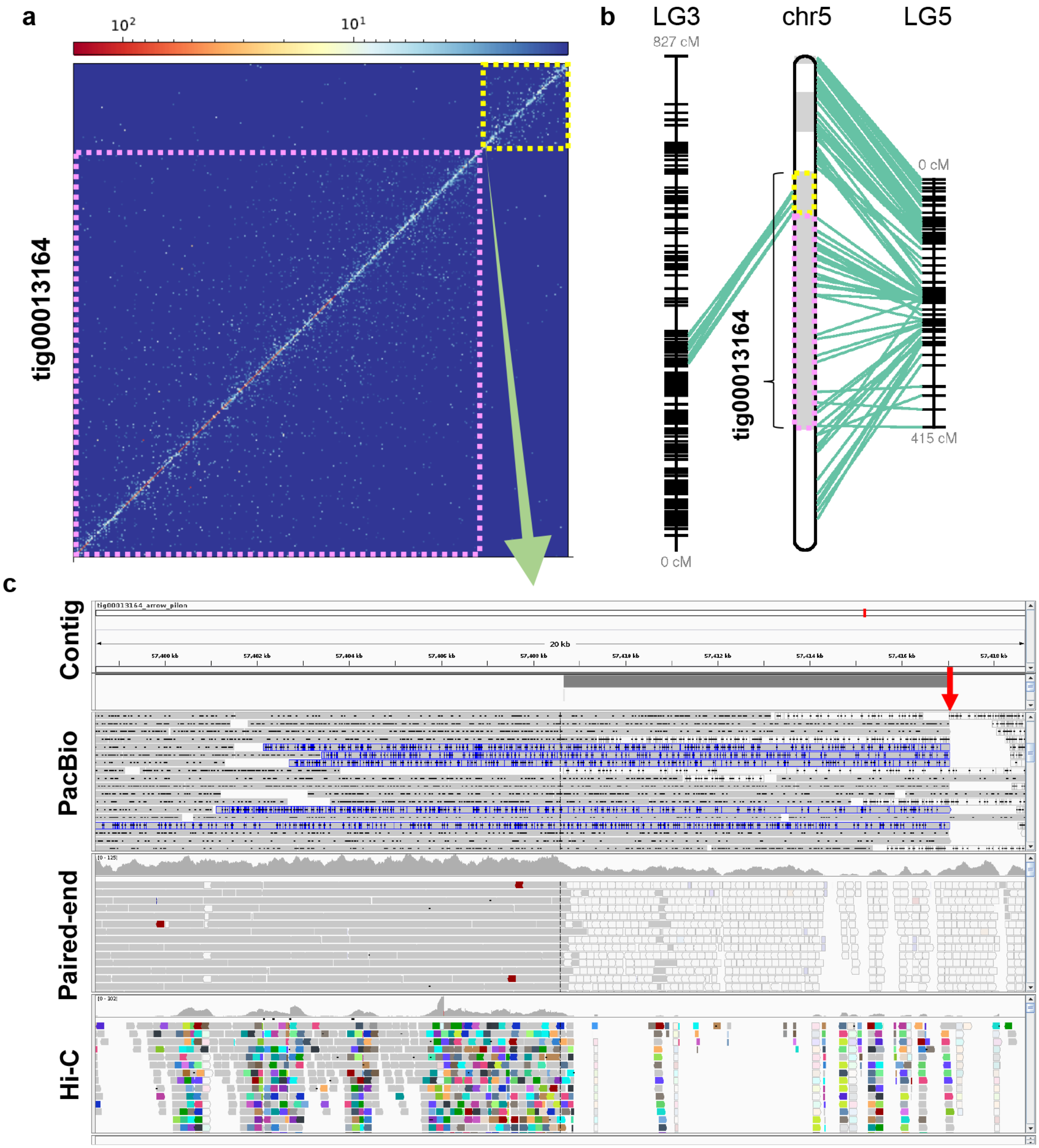

Supplementary Fig. 1. Example of a misassembled contig in the initial PacBio long read assembly. (**a**) Heatmap of Hi-C interactions of contig tig00013164, indicating a possible misjoining of two regions (marked with yellow and pink dashed-lines, respectively) indicated by very few Hi-C interactions. Color bar at the top represents the density of Hi-C interactions, which are indicated by number of links at the 50-kb resolution. The misassembly and the break point are supported by the evidence from genetic maps (**b**) and alignments of PacBio long reads, Illumina paired-end reads and Hi-C long contact reads (**c**) Most of the PacBio long reads were either ended or split (reads with blue border) at the suggested break point (pointed with red arrow).

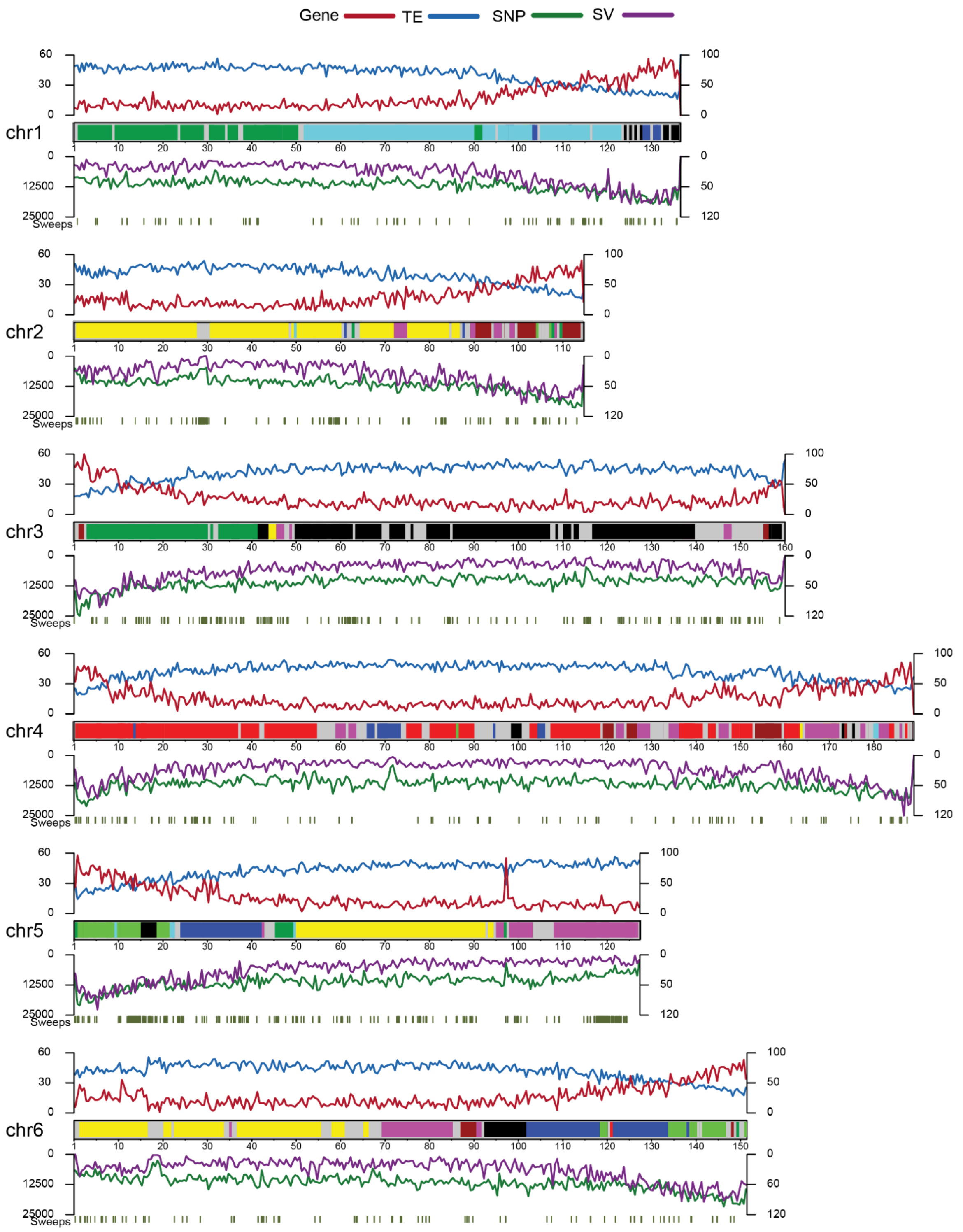

Supplementary Fig. 2. Genomic landscape of the six Monoe-Viroflay chromosomes. Gene density and SNP density are plotted using the left Y-axis while TE density and SV density are plotted using the right Y axis. All six chromosomes were painted based on the color of Chenopodiaceae ancestral karyotype shown in **Fig.2**.

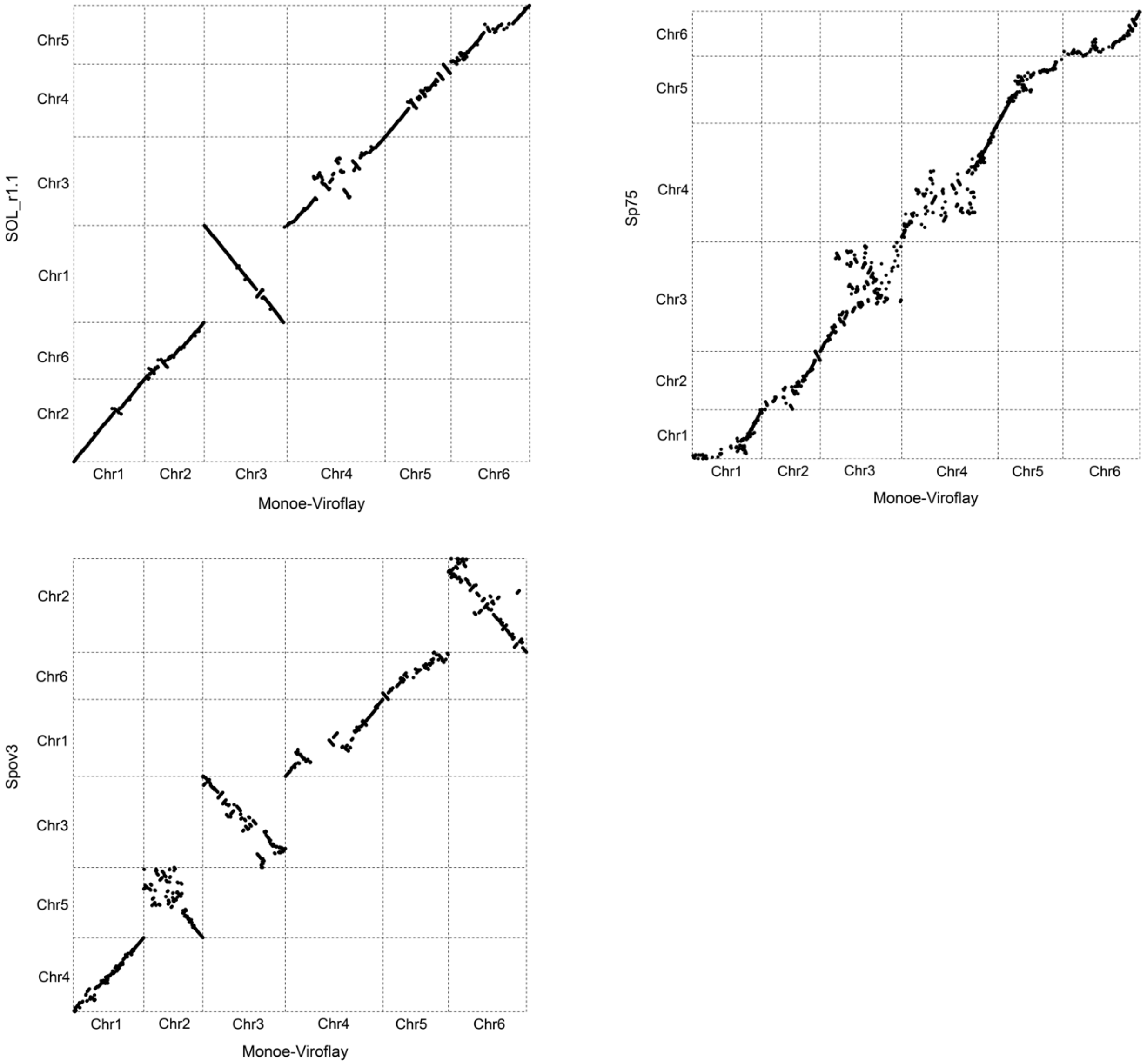

Supplementary Fig. 3. Alignments between the Monoe-Viroflay genome and the other three spinach genome assemblies (SOL_r1.1, Sp75 and Spov3). Chromosome names are adopted from the corresponding individual assemblies.

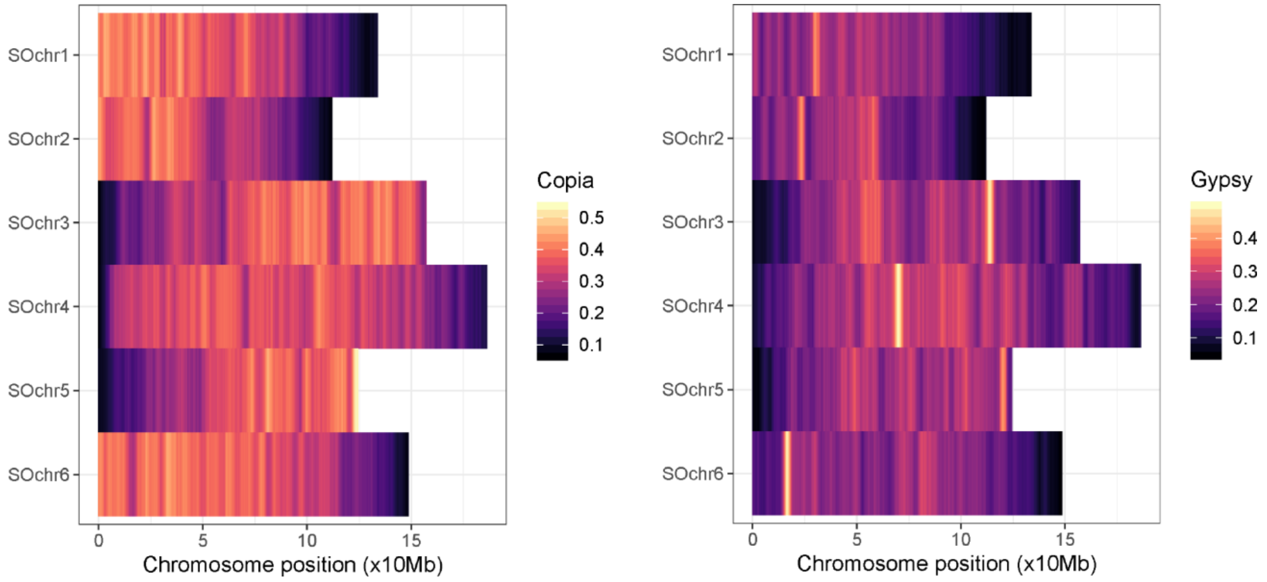

Supplementary Fig. 4. Distribution of *Copia* and *Gypsy*-type LTR retrotransposons in the Monoe-Viroflay genome.

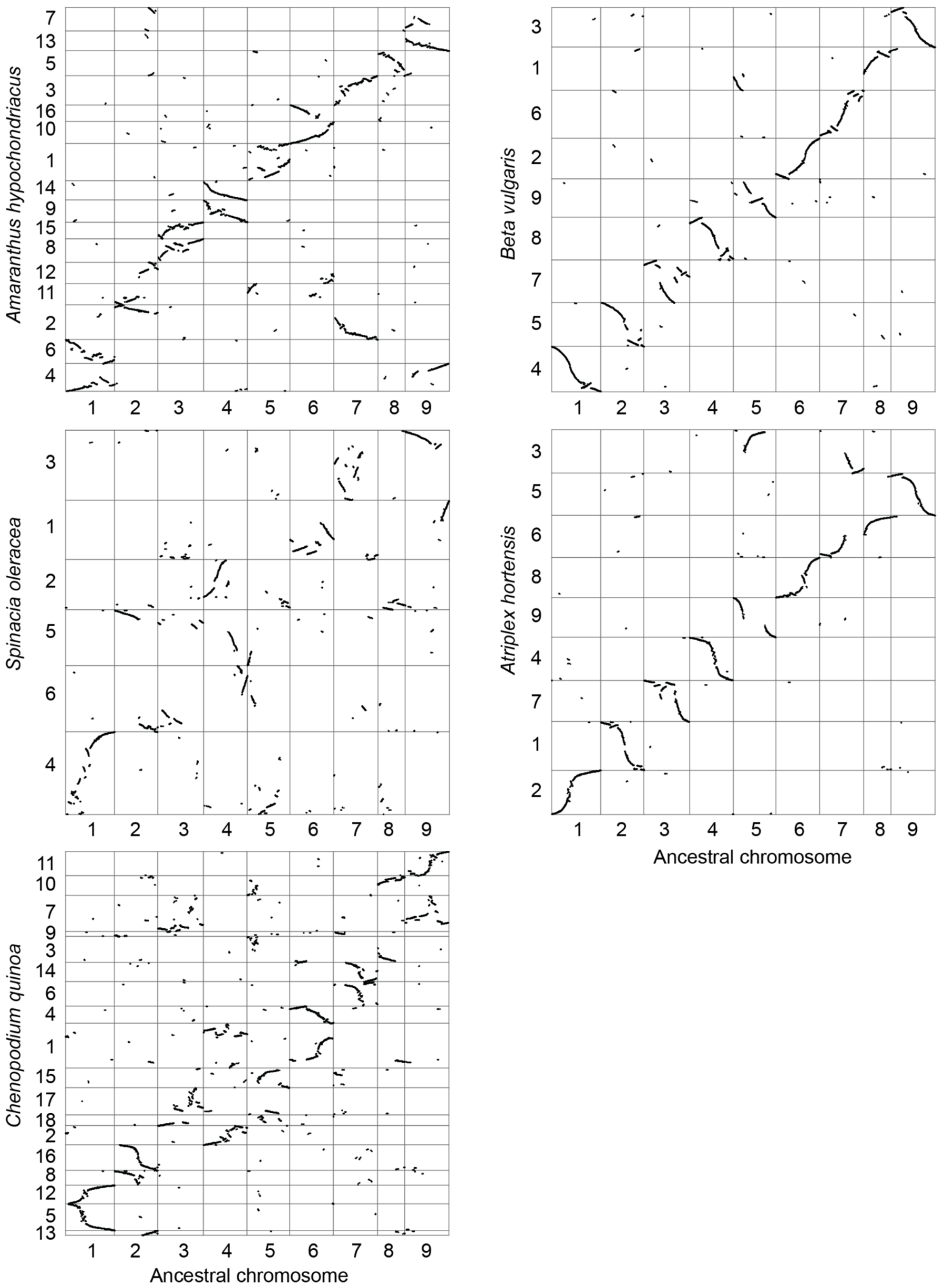

Supplementary Fig. 5. Genome synteny between extant and ancestral Chenopodiaceae.

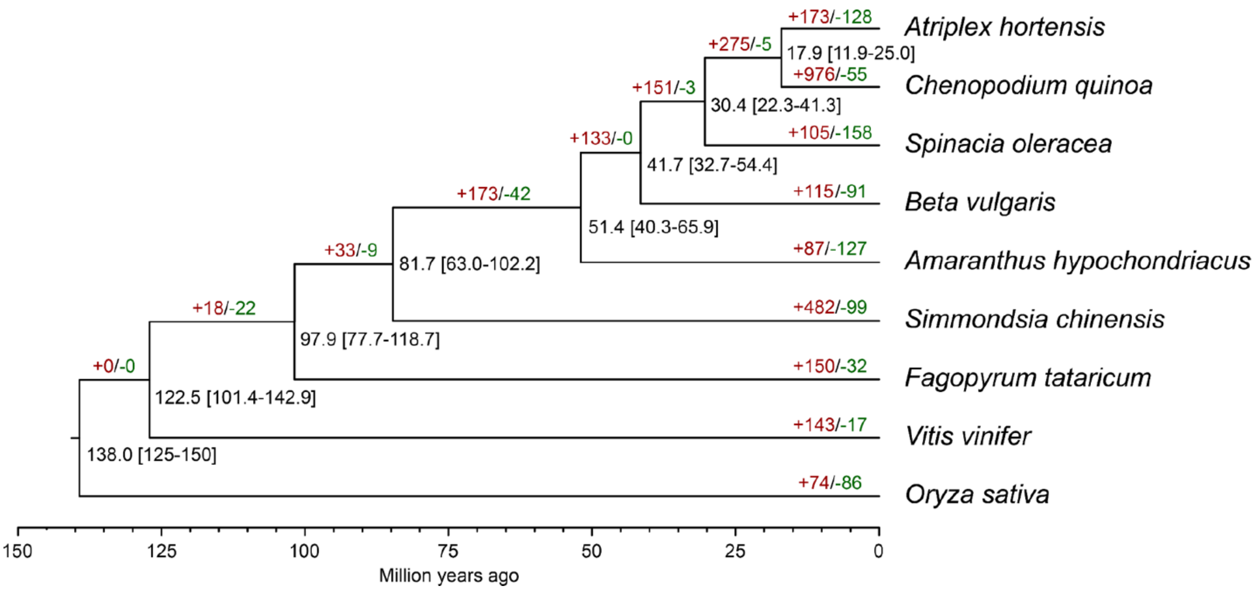

Supplementary Fig. 6. Phylogeny and gene family evolution of selected species. Black numbers around the branch of the tree represent the divergence time (million years ago) and the 95% highest posterior density range (in the bracket). Red and green numbers on the tree represent numbers of expanded (+) and contracted (-) gene families across the evolution of the species.

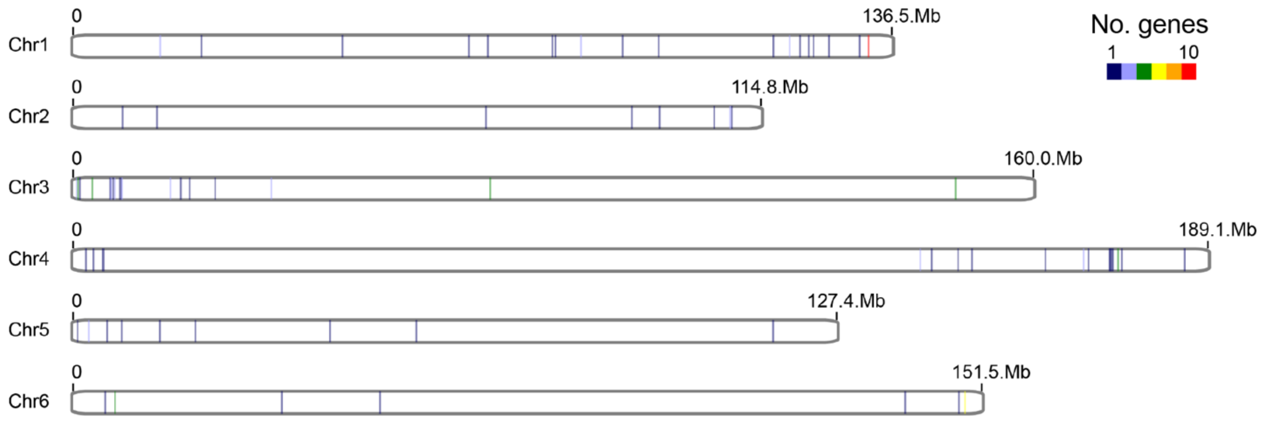

Supplementary Fig. 7. Distribution of NBS-LRR genes in the Monoe-Viroflay genome. The NBS-LRR gene clusters and singletons are plotted on each chromosome. The size of gene clusters is indicated by the color.

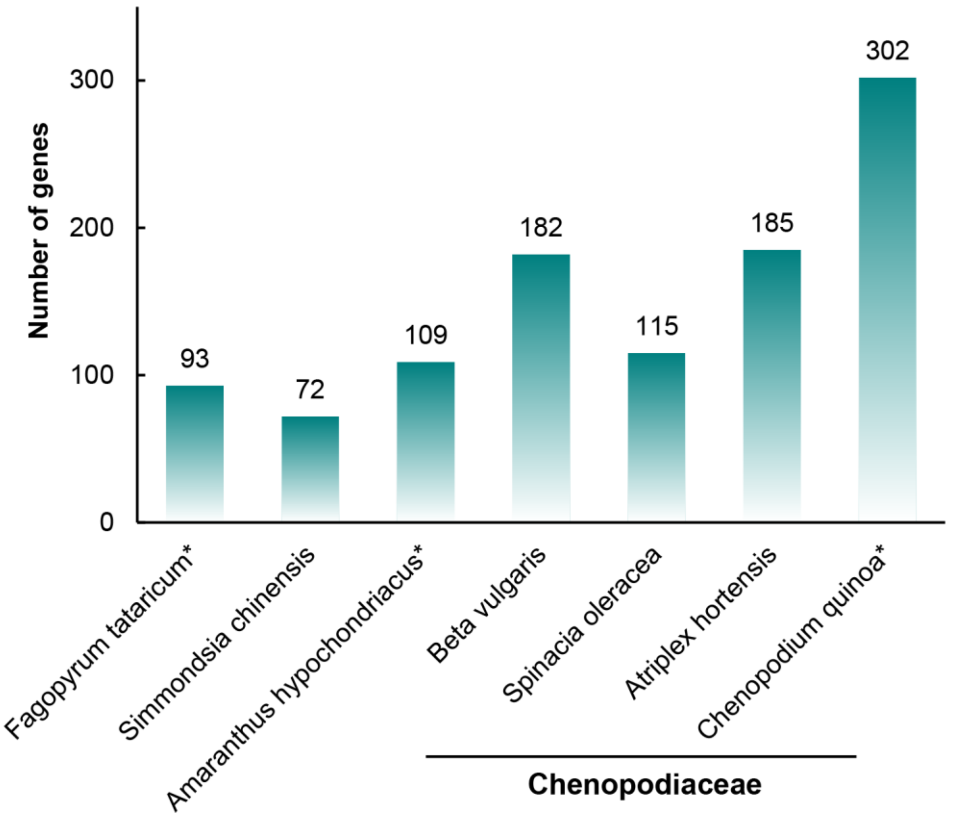

Supplementary Fig. 8. Number of NBS-LRR genes in the selected species.

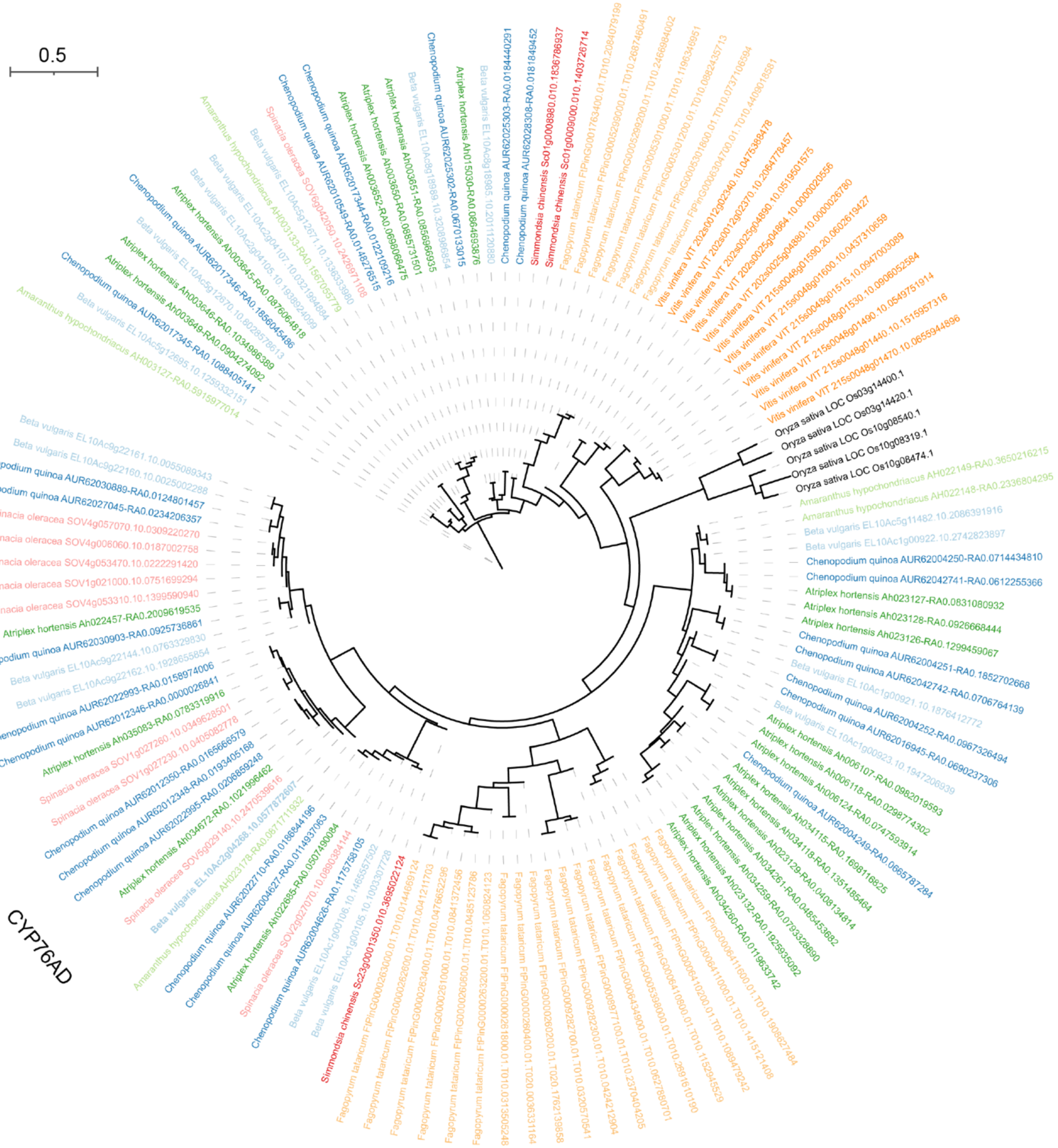

Supplementary Fig. 9. Phylogeny of CYP76AD genes in the selected species. Species are labeled in different color.

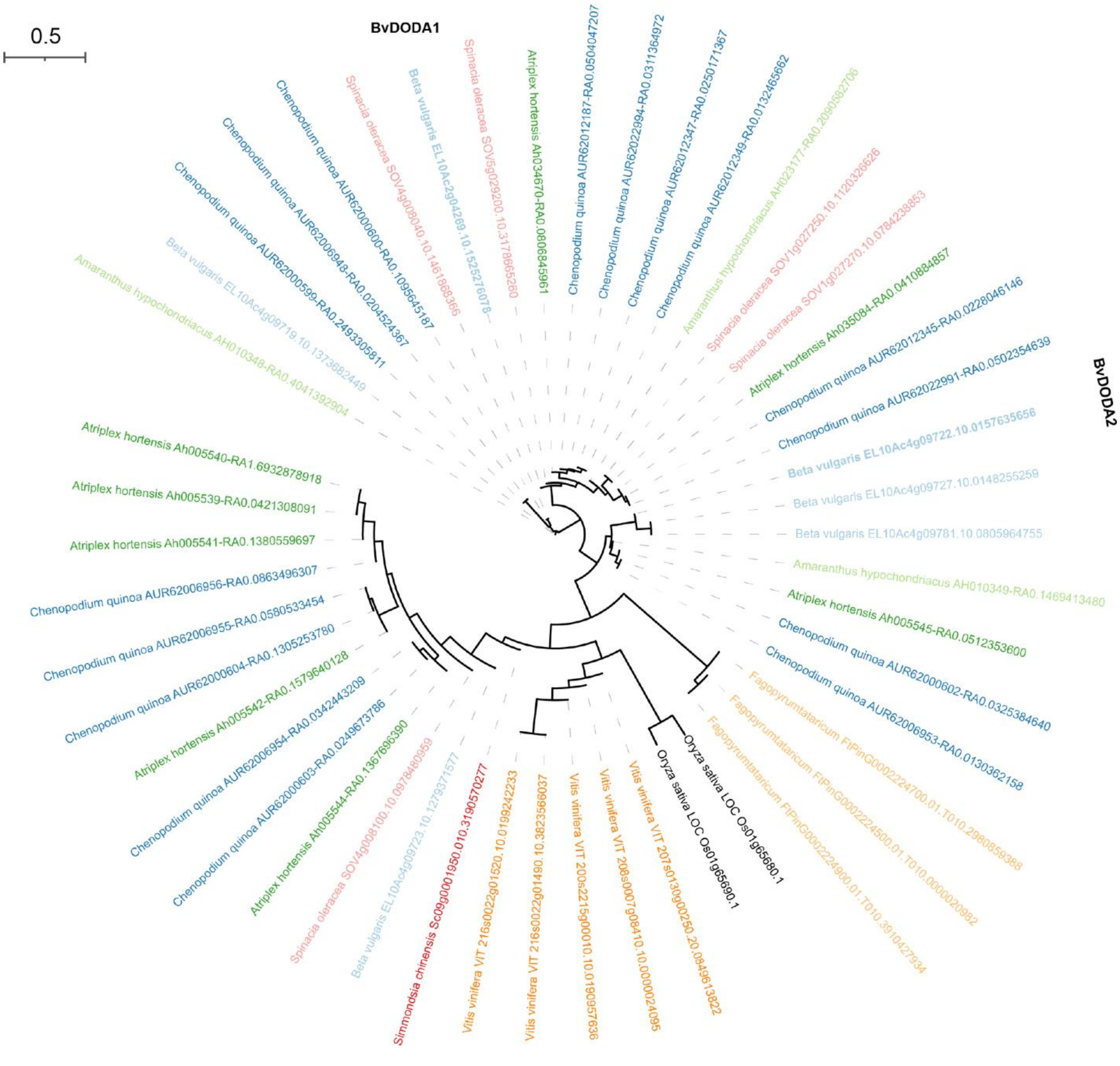

Supplementary Fig. 10. Phylogeny of DODA genes in the selected species. Species are labeled in different color.

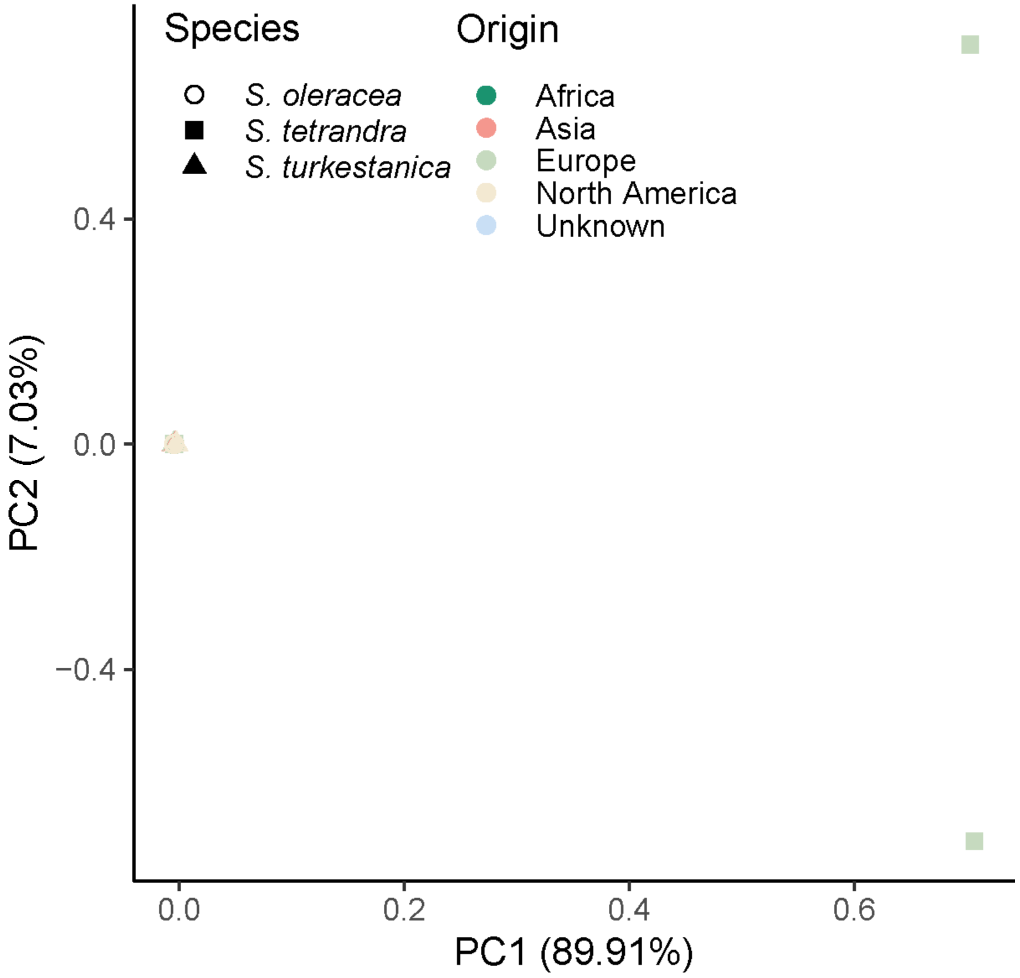

Supplementary Fig. 11. Principal component analysis of *Spinacia* accessions using SNPs at fourfold degenerate sites.

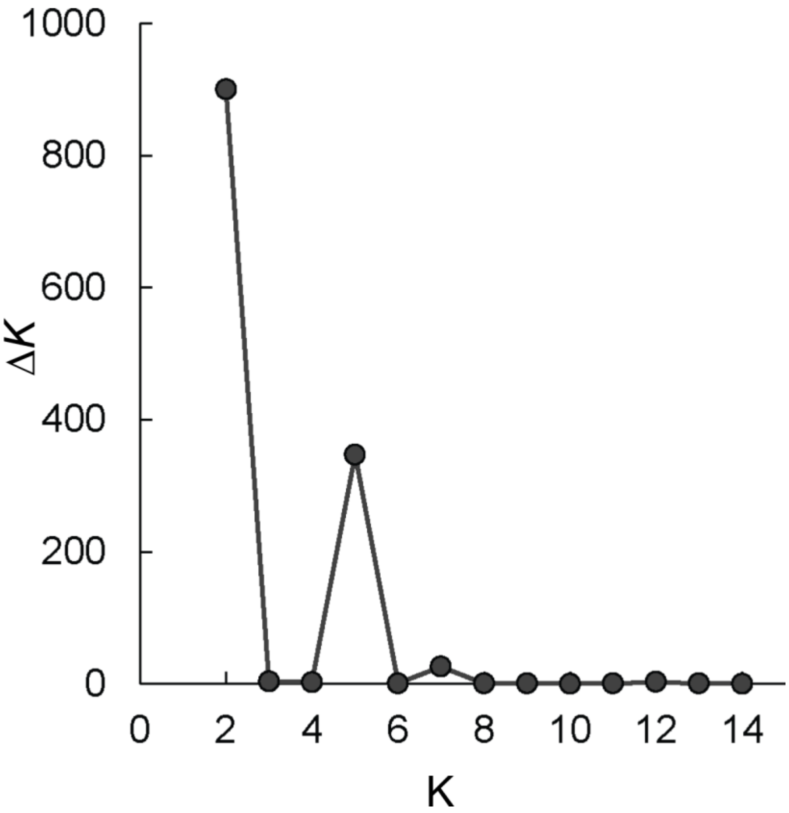

Supplementary Fig. 12. Estimated Δ*K* values with *K* from 2 to 14.

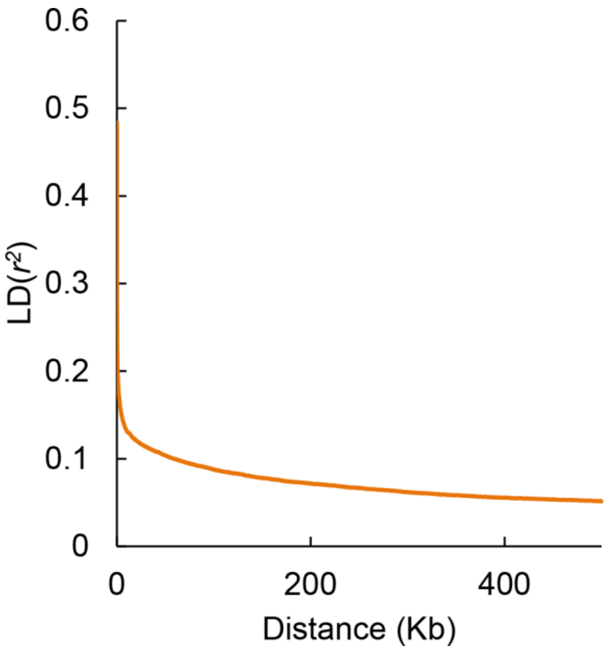

Supplementary Fig. 13. Linkage disequilibrium (LD) decay pattern of cultivated spinach (*Spinacia oleracea*).

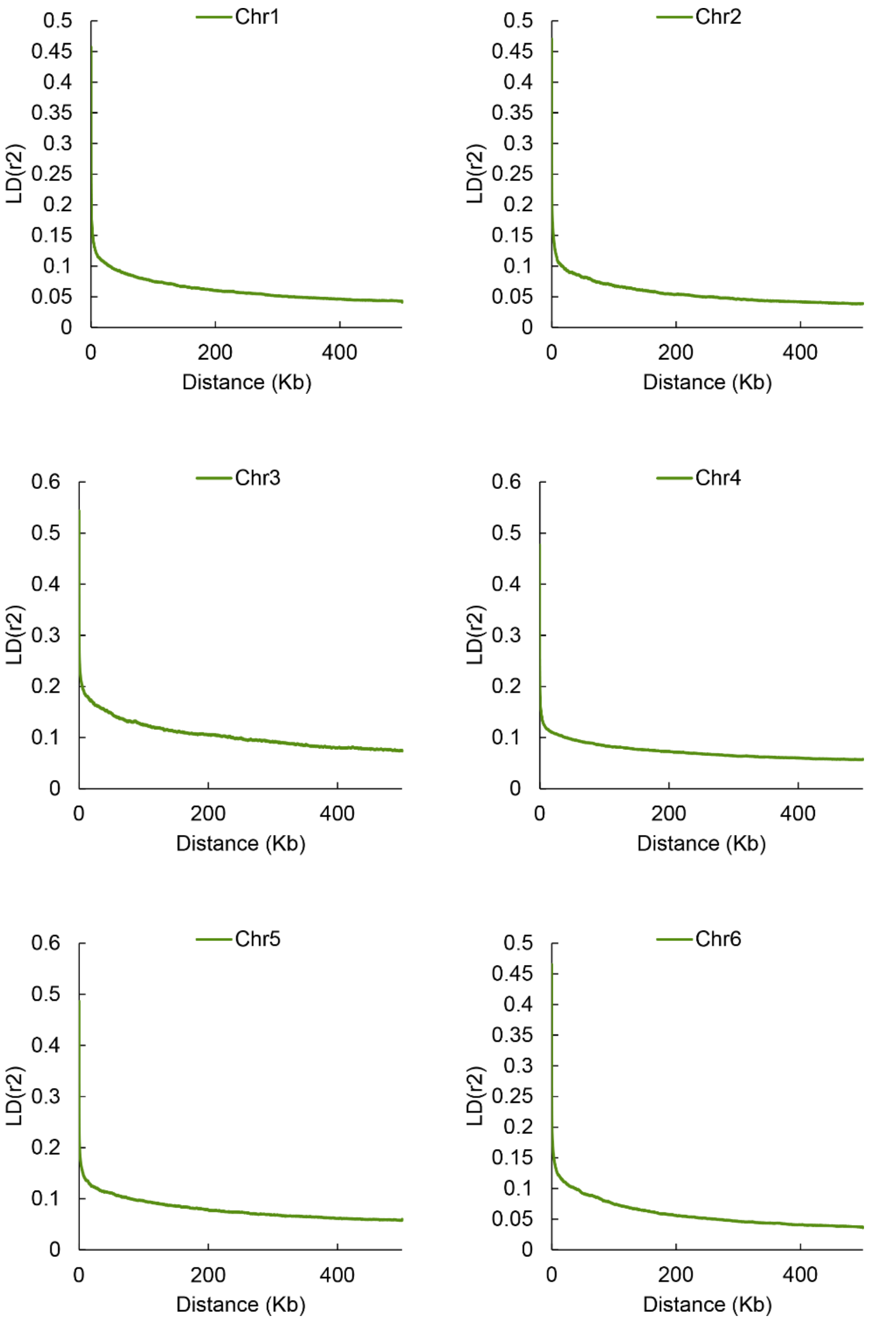

Supplementary Fig. 14. Linkage disequilibrium (LD) decay pattern of the six chromosomes of cultivated spinach (*Spinacia oleracea*).

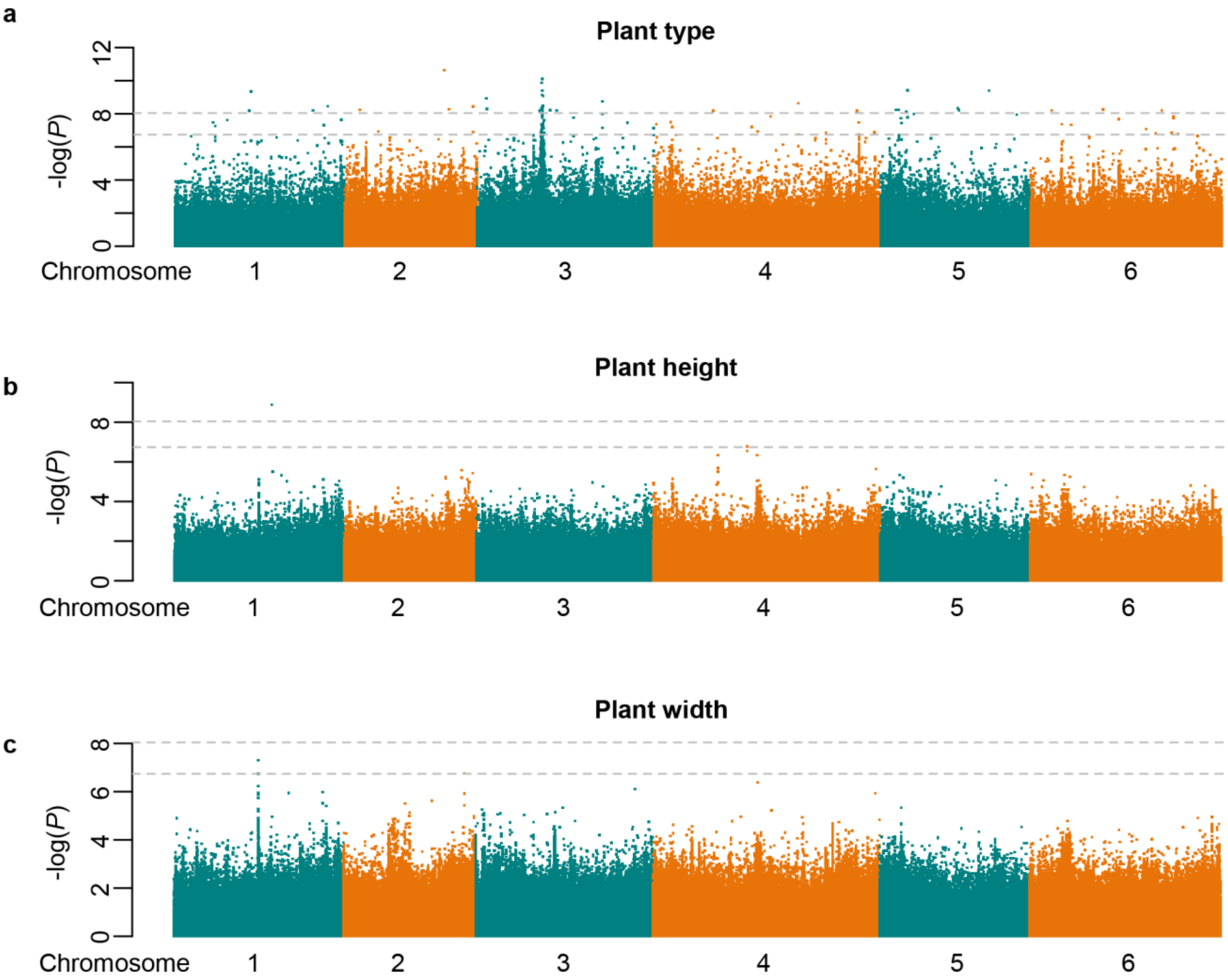

Supplementary Fig. 15. Manhattan plots of GWAS of plant type (a), plant height (b) and plant width (c). Gray horizontal dashed lines indicate the Bonferroni-corrected significance thresholds of GWAS (α = 0.05 and α = 1, respectively).

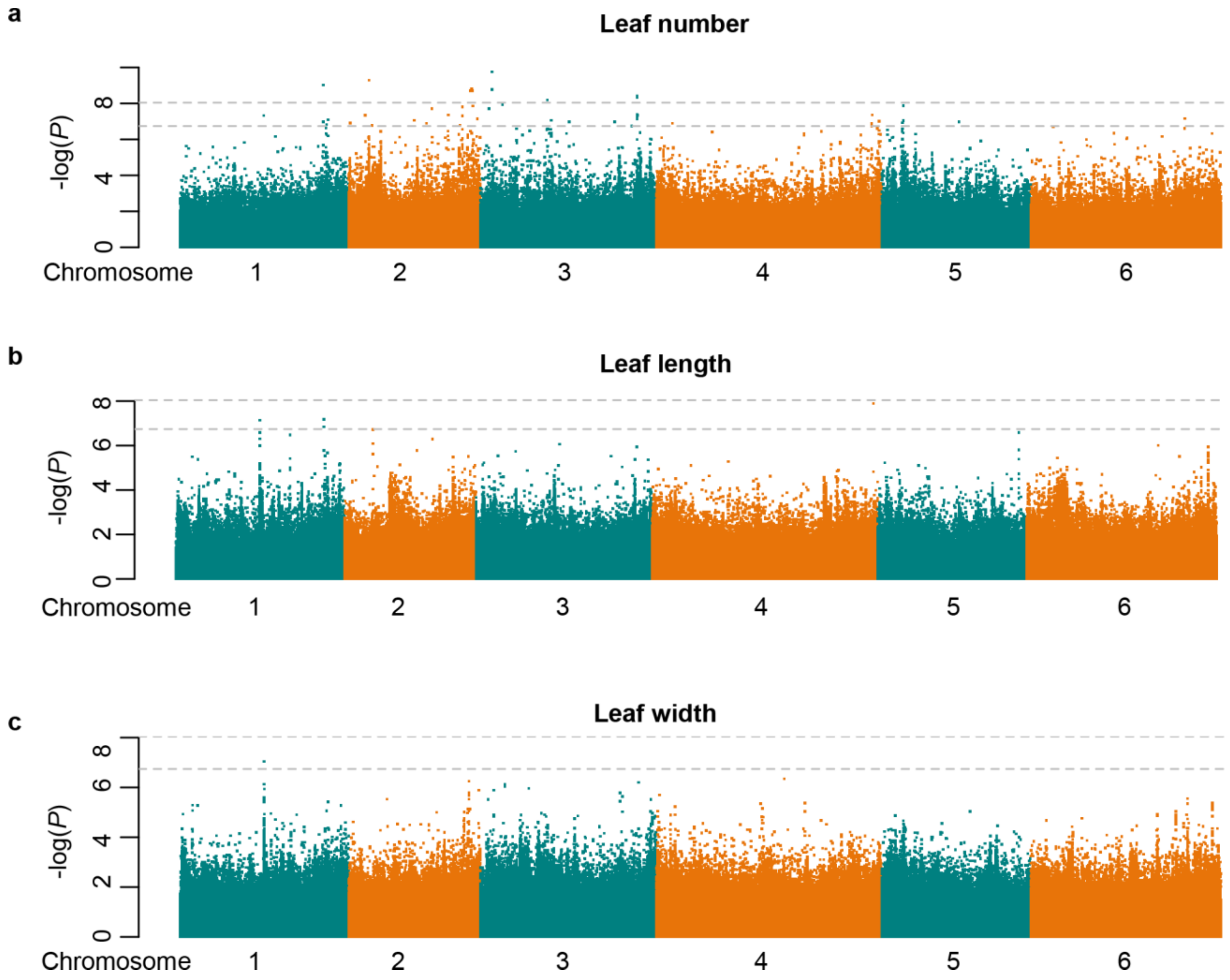

Supplementary Fig. 16. Manhattan plots of GWAS of leaf number(a), leaf length (b) and leaf width (c). Gray horizontal dashed lines indicate the Bonferroni-corrected significance thresholds of GWAS (α = 0.05 and α = 1, respectively).

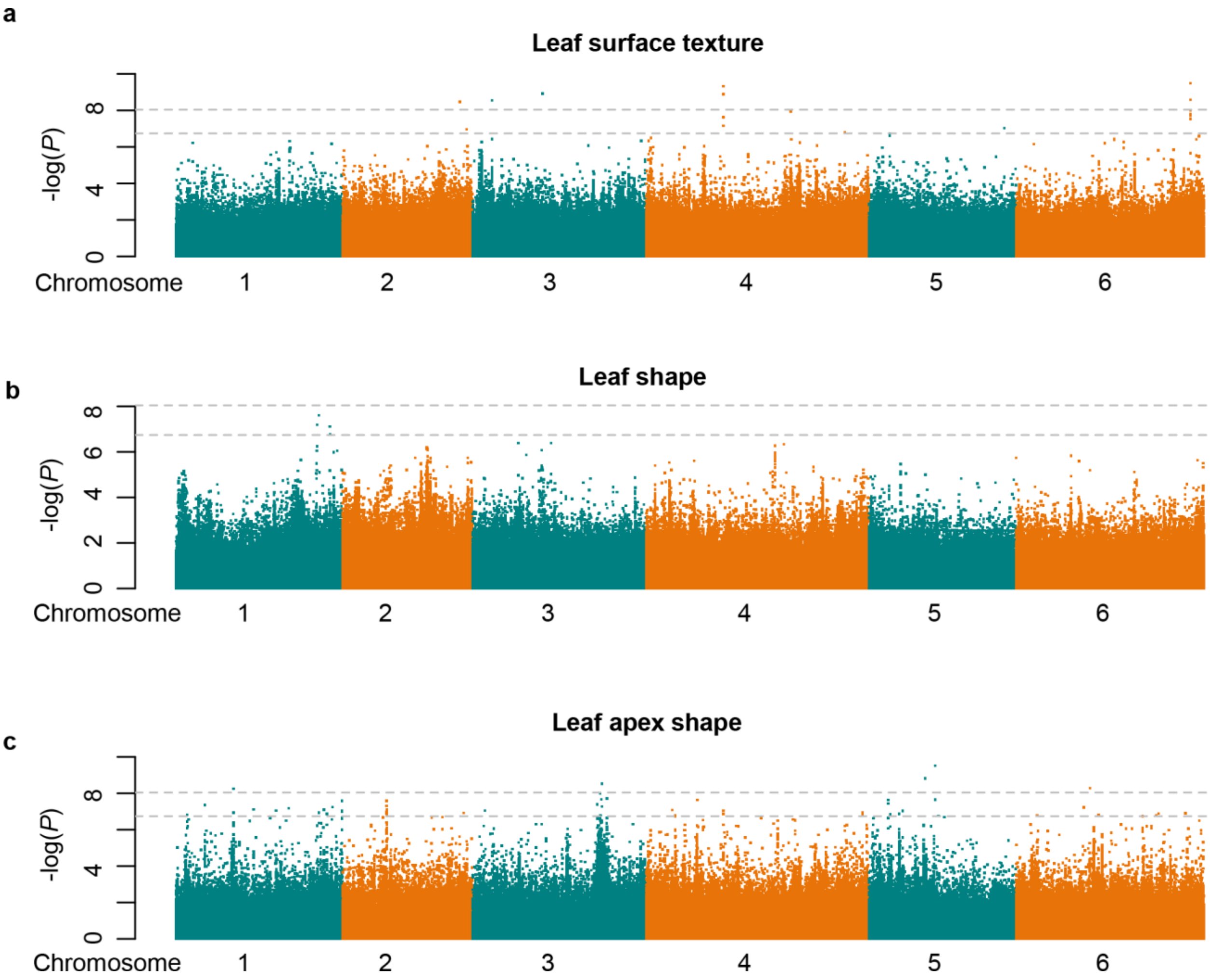

Supplementary Fig. 17. Manhattan plots of GWAS of leaf surface texture (a), leaf shape (b) and leaf apex shape (c). Gray horizontal dashed lines indicate the Bonferroni-corrected significance thresholds of GWAS (α = 0.05 and α = 1, respectively).

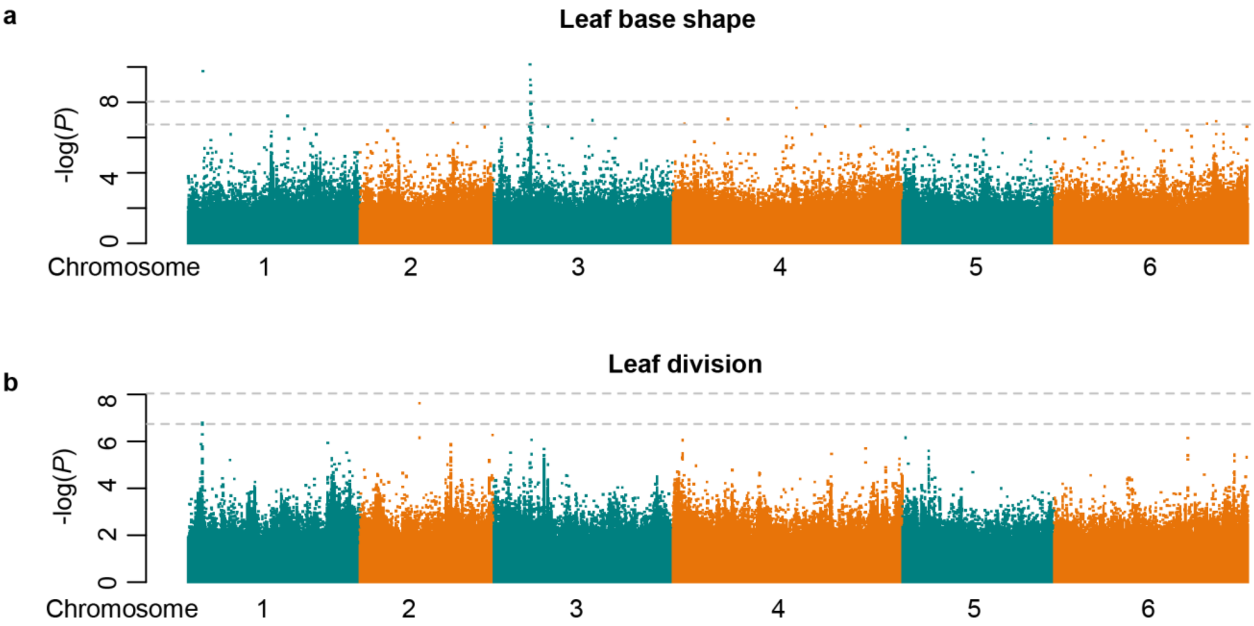

Supplementary Fig. 18. Manhattan plots of GWAS of leaf base shape (a) and leaf division (b). Gray horizontal dashed lines indicate the Bonferroni-corrected significance thresholds of GWAS (α = 0.05 and α = 1, respectively).

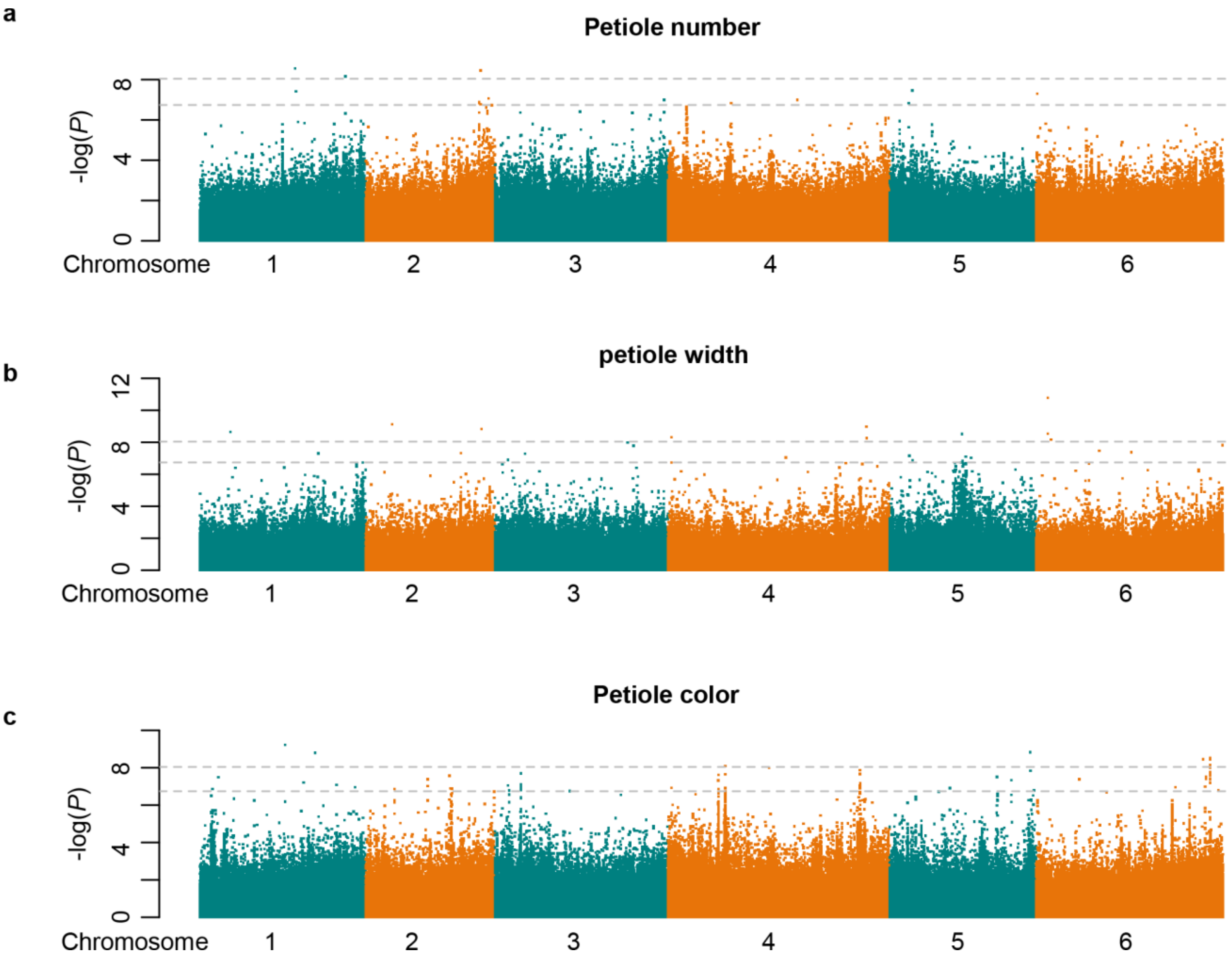

Supplementary Fig. 19. Manhattan plots of GWAS of petiole length (a), petiole width (b) and petiole color (c). Gray horizontal dashed lines indicate the Bonferroni-corrected significance thresholds of GWAS (α = 0.05 and α = 1, respectively).

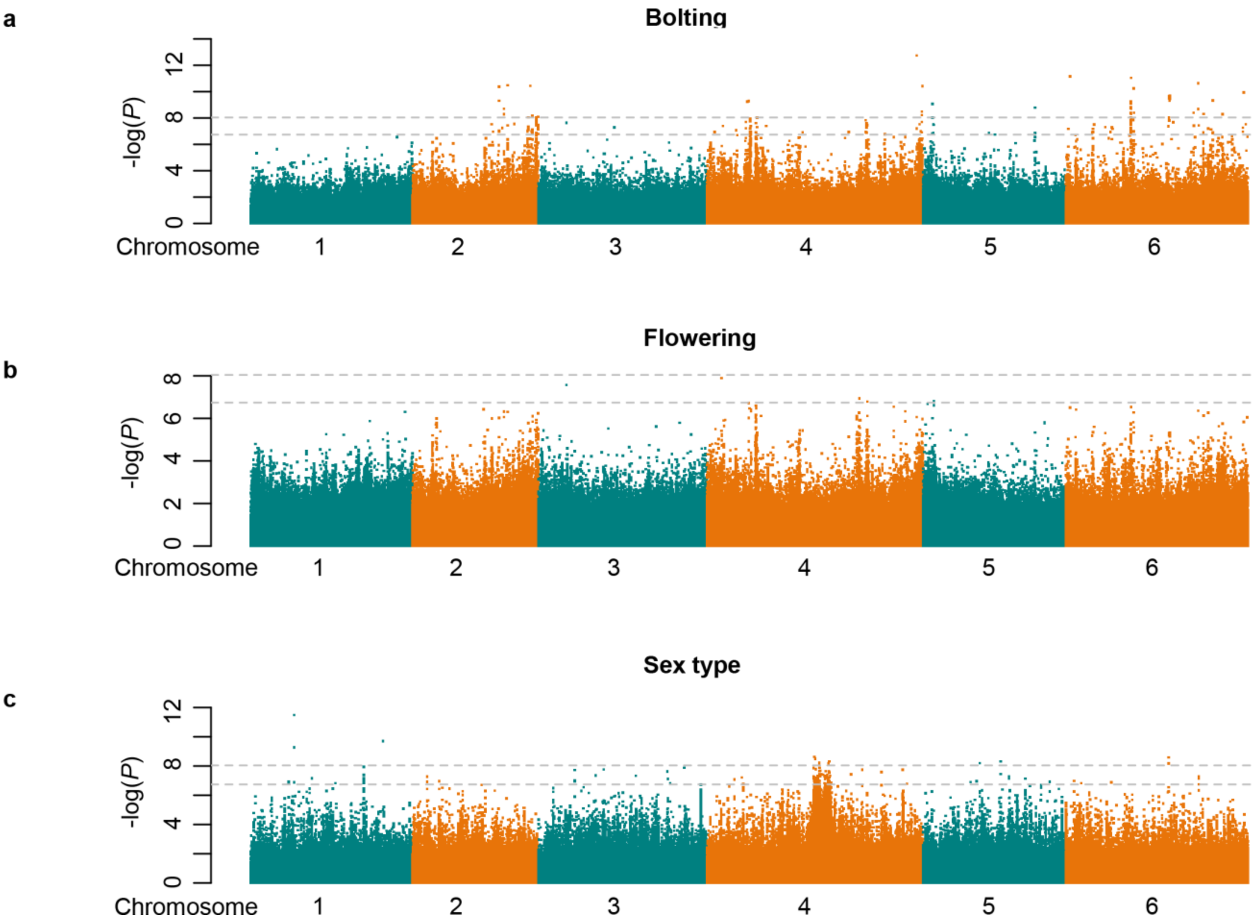

Supplementary Fig. 20. Manhattan plots of GWAS of bolting (a), flowering (b) and sex type (c). Gray horizontal dashed lines indicate the Bonferroni-corrected significance thresholds of GWAS ($\alpha = 0.05$ and $\alpha = 1$, respectively).

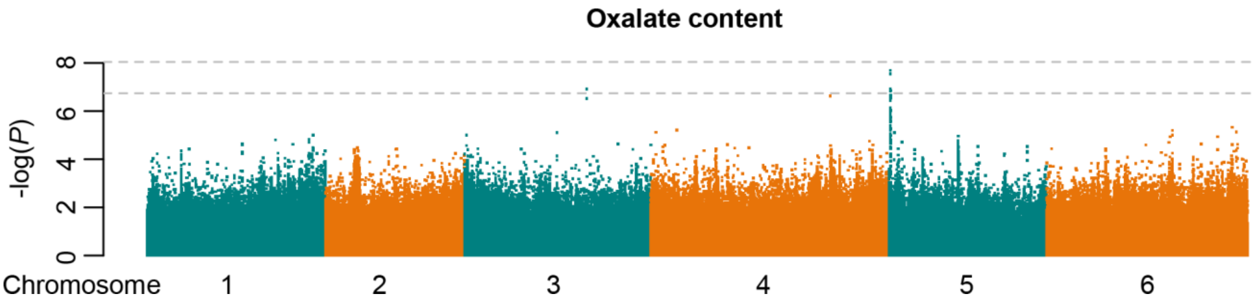

Supplementary Fig. 21. Manhattan plots of GWAS of oxalate content. Gray horizontal dashed lines indicate the Bonferroni-corrected significance thresholds of GWAS (α = 0.05 and α = 1, respectively).

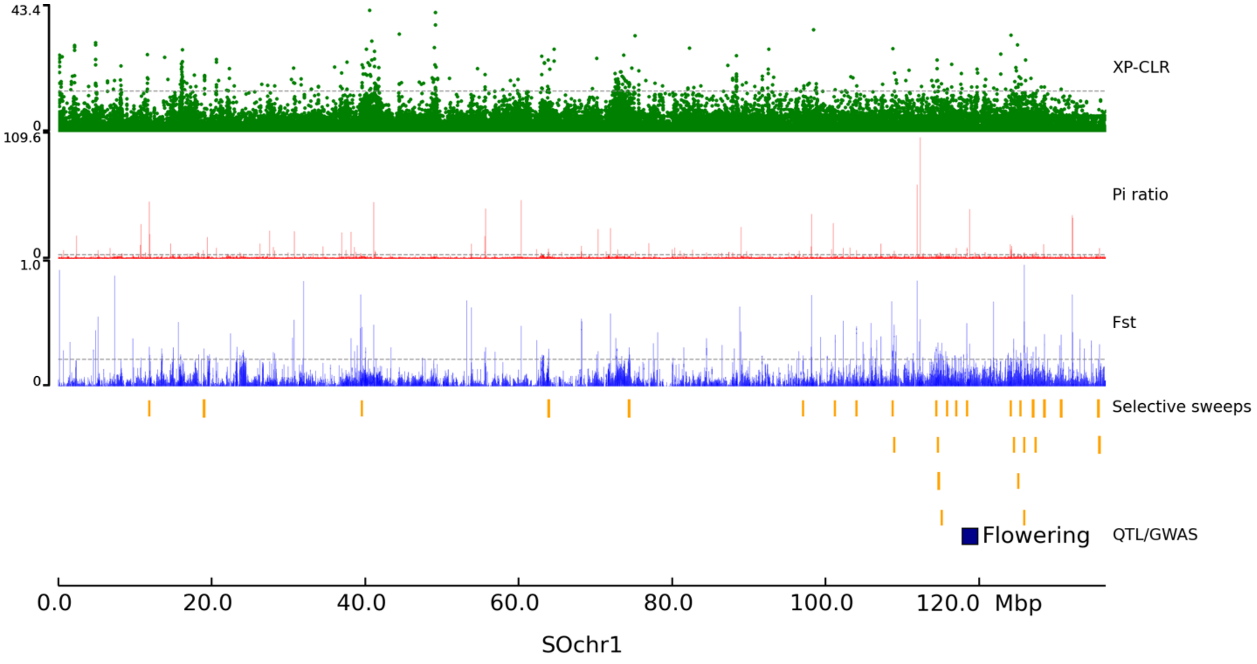

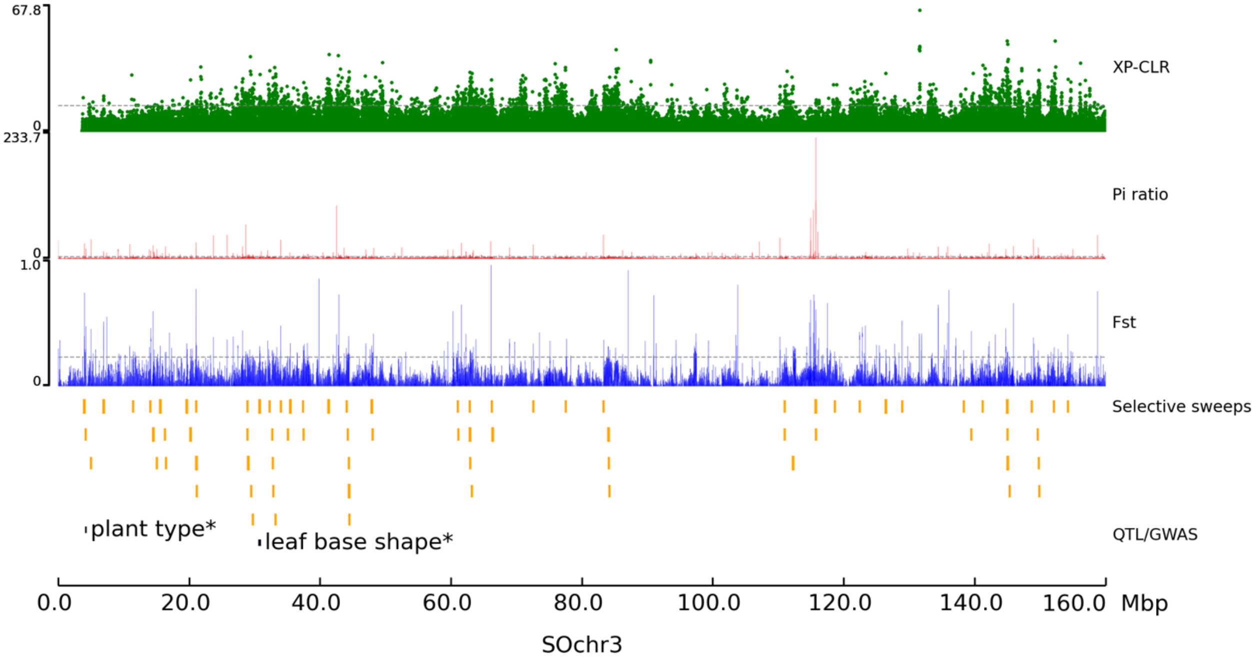

Supplementary Fig. 22. Genome-wide screening of selective sweeps during spinach domestication. Gray horizontal dashed lines indicate the top 1% thresholds of the scores/values derived from three approaches, XP-CLR, F_{ST} and nucleotide diversity (π) ratio. Putative selected regions (orange rectangles) and QTL/GWAS signals overlapping with the identified selective sweeps (black rectangles) are shown below these tracks. Traits with * indicate that corresponding GWAS signals were identified in this study.

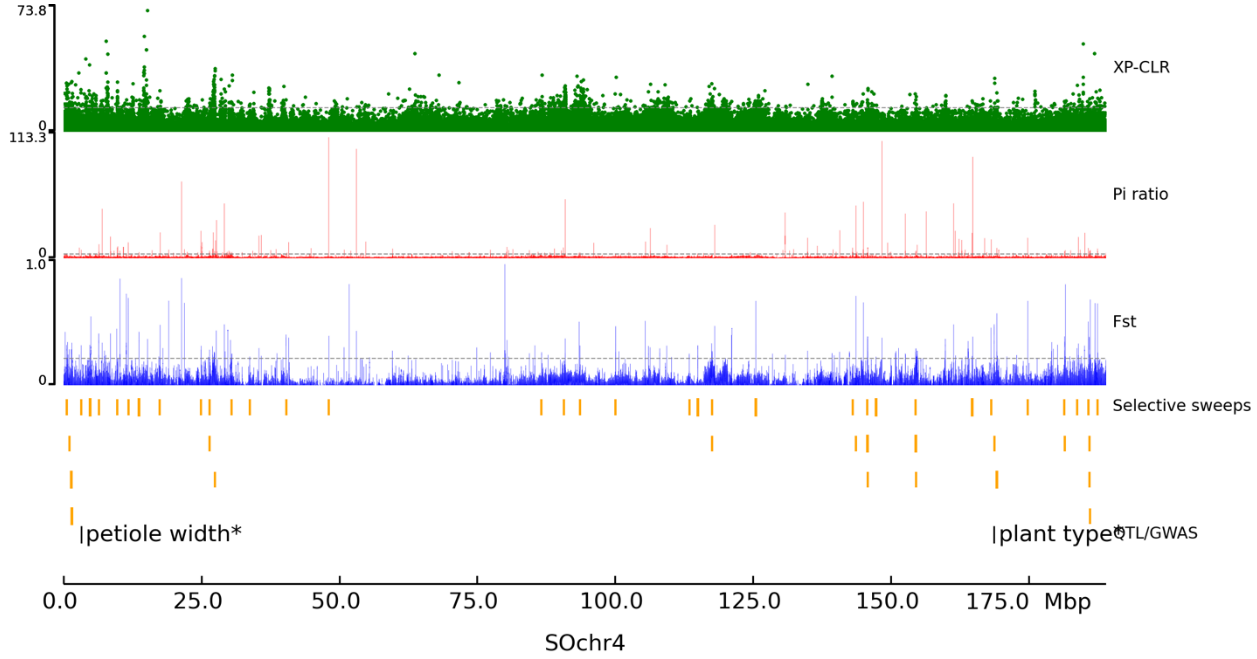

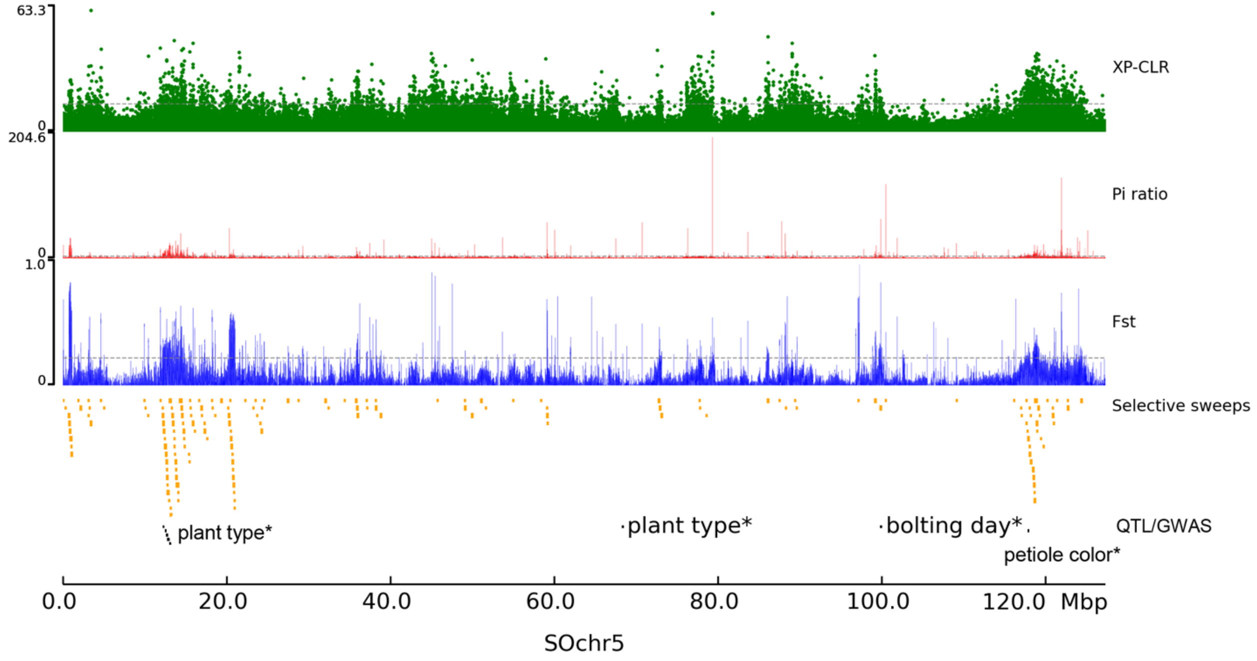

Supplementary Fig. 22 (continued)

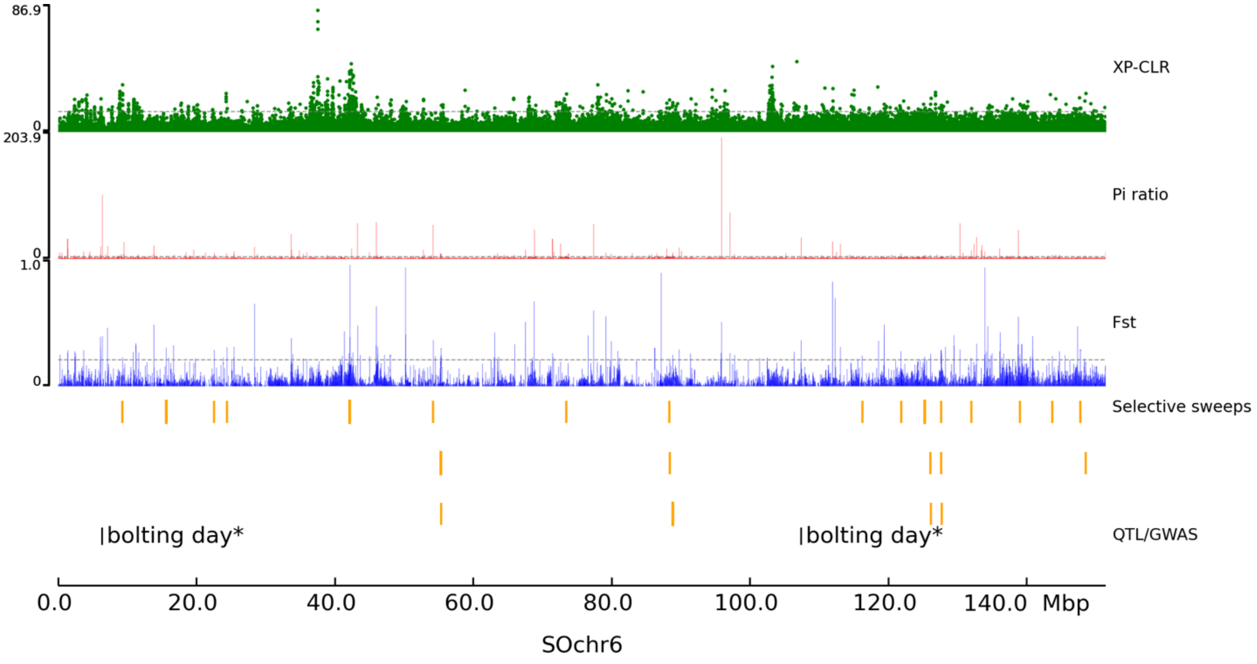

Supplementary Fig. 22 (continued)

Supplementary Table 1. Summary statistics of spinach Monoe-Viroflay genome sequencing.

Library type	No. Reads	No. base pairs	Read layout	Depth (x)
PacBio long reads	7,757,726	110,417,394,530	50 - 100,889	118
Illumina Paired-end reads	339,699,944	101,909,983,200	2X150	109
Dovetail™ Chicago®	208,485,612	62,545,683,600	2X150	67
Dovetail™ Hi-C	176,063,858	52,819,157,400	2X150	57

Supplementary Table 2. Quality evaluation of the Monoe-Viroflay genome assembly using Merqury (https://github.com/marbl/merqury).

Assembly	k-mers uniquely found in the assembly	k-mers found in both assembly and the read set	QV	Base error rate	Solid k-mers in the assembly	Total solid k-mers in the read set	Completeness (%)
Chr1	12,746	136,523,924	53.0857	4.91E-06			
Chr2	6,215	114,788,016	55.452	2.85E-06			
Chr3	14,472	160,042,660	53.2244	4.76E-06			
Chr4	29,645	189,049,996	50.8335	8.25E-06			
Chr5	14,705	127,366,691	52.1632	6.08E-06			
Chr6	15,009	151,449,199	52.8265	5.22E-06			
Chr0	314,321	15,024,006	29.5382	0.00111			
All	407,113	894,244,492	46.204	2.40E-05	311,989,010	313977520	99.3667

Supplementary Table 3. Genome evaluation using BUSCO and LAI.

	**Viroflay**	**Sp75**	**Spov3**	**SOL_r1.1**
Complete BUSCOs	1569 (97.2%)	1567 (97.1%)	1569 (97.2%)	1569 (97.2%)
Complete and single-copy BUSCOs (S)	1530 (94.8%)	1523 (94.4%)	1529 (94.7%)	1504 (93.2%)
Complete and duplicated BUSCOs (D)	39 (2.4%)	44 (2.7%)	40 (2.5%)	65 (4.0%)
Fragmented BUSCOs (F)	13 (0.8%)	16 (1.0%)	10 (0.6%)	13 (0.8%)
Missing BUSCOs (M)	32 (2.0%)	31 (1.9%)	35 (2.2%)	32 (2.0%)
LAI score	20.32	1.42	18.7	16.14

A total of 1614 embryophyta BUSCO groups were used for the analysis.

Supplementary Table 4. Repeat sequences in the Monoe-Viroflay genome assembly.

Class			Count	No. base masked	%masked
DNA	TIR	CMC-EnSpm	16,444	10,689,634	1.20%
		MULE-MuDR	4,502	4,166,811	0.47%
		PIF-Harbinger	202	248,931	0.03%
		TcMar-Mogwai	18	14,103	0.00%
		TcMar-Stowaway	1,544	1,098,965	0.12%
		hAT-Ac	984	1,011,162	0.11%
		hAT-Tag1	132	131,964	0.01%
		hAT-Tip100	95	125,321	0.01%
		MITE	44,370	11,808,772	1.32%
	Helitron		306	470,976	0.05%
RNA	LINE	CRE-II	1,524	1,100,784	0.12%
		L1	17,800	18,972,572	2.12%
		RTE-BovB	3,013	538,205	0.06%
	LTR	Caulimovirus	5	9,408	0.00%
		Copia	172,397	238,849,314	26.71%
		ERVK	42	129,832	0.01%
		Gypsy	93,895	152,343,918	17.04%
		other	95,076	118,681,027	13.27%
Unknown			45,924	18,828,481	2.11%
Low complexity			23,985	1,288,817	0.14%
Simple repeat			167,608	44,270,354	4.95%
Total			689,866	624,779,351	69.87%

Supplementary Table 5. Function annotation of predicted genes.

Database	No.	%
TAIR	22,641	78.17
Swiss-Prot	18,939	65.39
TrEMBL	26,986	93.17
InterPro	19,662	67.88
Pfam	18,909	65.28
Gene Ontology	20,489	70.74
Enzyme Commission	3,580	12.36
KEGG	8,201	28.31

Supplementary Table 6. Summary statistics of the identified variants in the spinach population.

	No. raw variants	After hard filtering	MinQ of 60 & Missing < 0.5	MinQ of 60 & Missing <0.5 & MAF > 0.01
**SNPs**	28,141,149	20,219,102	17,760,485	5,511,663*
**SV**	94,210	90,599 (MinQ 60)	68,328	55,330

*data was used in the GWAS analyses.

	Excluded sample	No. samples	No. SNPs
S. tetrandra	US351	2	3,777,933
S. turkestanica	US359	6	4,072,013
S. oleracea	US322 & USX	293	9,714,930
S. oleracea & S. turkestanica	US322 & USX & US359	299	10,041,507
S. oleracea & S. tetrandra	US322 & USX & US351	295	17,323,995
S. turkestanica & S. tetrandra	US351 & US359	8	12,030,147

Supplementary Table 7. Predicted impact of the SNPs.

Impact severity	Catergories in SnpEff	No.	%
low	intergenic	8,852,842	49.85
	downstream	2,266,232	12.76
	intron	2,256,006	12.70
	synonymous coding	481,097	2.71
	synonymous start	1	0.00
	synonymous stop	725	0.00
	upstream	3,049,144	17.17
	UTR 3 prime	220,625	1.24
	UTR 5 prime	116,463	0.66
medium	non synonymous coding	475,216	2.68
high	non synonymous start	169	0.00
	splice site acceptor	3,707	0.02
	splice site donor	2,854	0.02
	start gained	18,977	0.11
	start lost	978	0.01
	stop gained	14,234	0.08
	stop lost	1,215	0.01

Supplementary Table 8. Nucleotide diversity and population differentiation.

	Species/groups	Mean
Nucleotide diversity π	*S. oleracea*	0.001325
	S. oleracea Asia	0.001543
	S. oleracea Europe	0.001229
	S. turkestanica	0.001521
Population fixation index F_{ST}	*S. oleracea* vs. *S. turkestanica*	0.031010
	S. oleracea Asia vs. *S. turkestanica*	0.043212
	S. oleracea Europe vs. *S. turkestanica*	0.051572
	S. oleracea Asia vs. *S. oleracea* Europe	0.058284

Supplementary Table 9. Summary statistics of each of the 20 traits in the spinach germplasm collection.

Trait	Minimum	Maximum	Mean ± SE	Standard deviation	Coefficient of variation (%)
Plant type	-	-	-		-
Plant height	1.7	31.5	13.396	4.569	34.10
Plant width	10.63	60.97	30.605	8.358	27.31
Leaf length	3.67	22.13	11.035	2.632	23.85
Leaf width	1.6	13.5	7.415	1.908	25.73
Leaf number	7	34	13.729	4.157	30.28
Leaf surface texture	-	-	-	-	-
Leaf shape	-	-	-	-	-
Leaf apex shape	-	-	-	-	-
Leaf base shape	-	-	-	-	-
Leaf division	-	-	-	-	-
Petiole length	1.53	19.87	7.669	2.806	36.59
Petiole width	0.1	1.1	0.343	0.13	37.90
Petiole color	-	-	-	-	-
Bolting days	40	203	138.6	18.1	13.06
Flowering days	93	230	162.51	15.853	9.76
Sex type	-	-	-	-	-
Oxalate content	0.59	1.93	1.334	0.321	24.06
DM resistance	-	-	-	-	-
DM incidence	0	100	54.335	27.818	51.20

Supplementary Table 10. Summary of the identified association signals.

Traits		$-\log_{10}(P)$ of the peak SNP	α	No. signals
Plant	plant type	10.62796	0.05	64
	plant height	8.87798	0.05	1
	plant width	7.29688	1	2
Leaf	leaf length	7.88883	1	4
	leaf width	7.03873	1	1
	leaf number	9.74964	0.05	25
	leaf surface texture	9.48179	0.05	11
	leaf shape	7.60211	1	4
	leaf apex shape	9.51058	0.05	6
	leaf base shape	10.13736	0.05	18
	leaf division	7.62169	1	2
Petiole	petiole length	8.54939	0.05	3
	petiole width	10.77373	0.05	11
	petiole color	9.21614	0.05	1
Other	bolting day	12.73674	0.05	82
	flowering day	7.88730	1	5
	oxalate content	7.68595	1	12
	sex type	11.48056	0.05	13
DM	DM resistance	11.79569	0.05	107
	DM incidence	7.84698	1	4

Supplementary Table 11. Summary Statistic of the selective sweeps.

Top1% regions						
**Methods**	**No.**	**Total size**	**Mean window size**	**Median window size**	**Min. window size**	**Max. window size**
F_{ST}	1,256	21,245,000	16,915	12,000	10,000	311,000
π	1,612	24,359,000	15,111	11,000	10,000	185,000
XP-CLR	5,397	9,544,265	1,768	833	1	77,728
**Overlapped windows by different methods**						
π & XP-CLR	418	9,162,000	21,919	15,000	10,000	185,000
F_{ST} & XP-CLR	346	8,338,000	24,098	18,000	10,000	311,000
π & F_{ST}	667	12,555,000	18,823	13,000	10,000	185,000
π & XP-CLR or π & F_{ST}	866	15,502,000	17,901	13,000	10,000	185,000
Any two method	996	17,621,000	17,692	13,000	10,000	185,000
