# Supplementary material for: Genomic analyses provide insights into spinach domestication and the genetic basis of agronomic traits
Source: Nat Commun. 2021 Dec 13;12:7246. doi: 10.1038/s41467-021-27432-z (PMC8668906; doi:10.1038/s41467-021-27432-z)
Supplement: Supplementary file 3 — Description of Additional Supplementary Files [file 41467_2021_27432_MOESM3_ESM.pdf]

Description of Additional Supplementary Files

Supplementary Data 1
Summary statistics of spinach genome assemblies

Supplementary Data 2
Contracted gene families

Supplementary Data 3
Expanded gene families

Supplementary Data 4
R genes in the Monoe_Viroflay genome

Supplementary Data 5
List of the 305 resequenced spinach accessions

Supplementary Data 6
Phenotypic data of 20 spinach traits for 303 spinach accession

Supplementary Data 7
Correlative coefficient of 20 phenotypic trait

Supplementary Data 8
Genes overlapped with the associated regions of different traits

Supplementary Data 9
Predicted effects of the associated signals of different traits

Supplementary Data 10
Genes in the putative selective sweep regions
