# Supplementary material for: Genomic analyses provide insights into spinach domestication and the genetic basis of agronomic traits
Source: Nat Commun. 2021 Dec 13;12:7246. doi: 10.1038/s41467-021-27432-z (PMC8668906; doi:10.1038/s41467-021-27432-z)
Supplement: Supplementary file 14 — Reporting Summary [file 41467_2021_27432_MOESM14_ESM.pdf]

nature portfolio

Corresponding author(s): Quanhua Wang, Chen Jiao, Zhangjun Fei, Quanxi Wang

Last updated by author(s): Nov 18, 2021

Reporting Summary

Nature Portfolio wishes to improve the reproducibility of the work that we publish. This form provides structure for consistency and transparency in reporting. For further information on Nature Portfolio policies, see our Editorial Policies and the Editorial Policy Checklist.

Statistics

For all statistical analyses, confirm that the following items are present in the figure legend, table legend, main text, or Methods section.

n/a	Confirmed	
☐	☒	The exact sample size (*n*) for each experimental group/condition, given as a discrete number and unit of measurement
☐	☒	A statement on whether measurements were taken from distinct samples or whether the same sample was measured repeatedly
☐	☒	The statistical test(s) used AND whether they are one- or two-sided<br>*Only common tests should be described solely by name; describe more complex techniques in the Methods section.*
☒	☐	A description of all covariates tested
☐	☒	A description of any assumptions or corrections, such as tests of normality and adjustment for multiple comparisons
☐	☒	A full description of the statistical parameters including central tendency (e.g. means) or other basic estimates (e.g. regression coefficient) AND variation (e.g. standard deviation) or associated estimates of uncertainty (e.g. confidence intervals)
☒	☐	For null hypothesis testing, the test statistic (e.g. *F*, *t*, *r*) with confidence intervals, effect sizes, degrees of freedom and *P* value noted<br>*Give P values as exact values whenever suitable.*
☒	☐	For Bayesian analysis, information on the choice of priors and Markov chain Monte Carlo settings
☒	☐	For hierarchical and complex designs, identification of the appropriate level for tests and full reporting of outcomes
☒	☐	Estimates of effect sizes (e.g. Cohen's *d*, Pearson's *r*), indicating how they were calculated

Our web collection on statistics for biologists contains articles on many of the points above.

Software and code

Policy information about availability of computer code

Data collection	No software was used in data collection.
Data analysis	We used freely available bioinformatics softwares, including: CANU (v1.7.1), Trimmomatic (v0.36), Pilon (v1.22), redundans(v0.14a), HiRise, MITE-Hunter (v11-2011), LTRharvest (v1.5.10), RepeatMasker(v4.0.8), RepeatModeler (v1.0.11), HISAT2 (v2.1), StringTie (v1.3.3b), PASA (v2.3.3), MAKER(v2.31.10), GeneMark-ES (v4.35), AUGUSTUS55 (v3.3), SNAP (v2006-07-28), MCScanX, MGRA (v2.3.0 beta), OrthoMCL (v2.0.9), MAFFT (v7.313), trimAl (v1.2), IQ-TREE (v1.6.7), MCMCTree, PAML (v4.8), CAFE (v 4.2), BWA-MEM(v0.7.17), GATK(v4.1), smoove, LUMPY (v0.2.13), SVTyper (v0.7.1), VCF2Dis, EIGENSOFT (v7.2.1), STRUCTURE (v2.3.4), PopLDdecay(v3.41), XP-CLR, VCFtools (v0.1.16), EMMAX (v20120210), bedtools (v2.26.0)

For manuscripts utilizing custom algorithms or software that are central to the research but not yet described in published literature, software must be made available to editors and reviewers. We strongly encourage code deposition in a community repository (e.g. GitHub). See the Nature Portfolio guidelines for submitting code & software for further information.

Data

Policy information about availability of data

All manuscripts must include a data availability statement. This statement should provide the following information, where applicable:

- Accession codes, unique identifiers, or web links for publicly available datasets
- A description of any restrictions on data availability
- For clinical datasets or third party data, please ensure that the statement adheres to our policy

e Genome Shotgun project has been deposited at DDBJ/ENA/GenBank under the accession WWFT00000000 (https://www.ncbi.nlm.nih.gov/nuccore/WWFT00000000). The version described in this paper is version WWFT01000000 (https://www.ncbi.nlm.nih.gov/nuccore/WWFT00000000.1). Raw genome and

transcriptome sequencing reads have been deposited in the NCBI BioProject database under the accession number PRJNA598728 (https://www.ncbi.nlm.nih.gov/bioproject/PRJNA598728). The 'Monoe-Viroflay' genome assembly and annotated genes, and SNPs and SVs in VCF file format are also available at SpinachBase (http://spinachbase.org/).

Field-specific reporting

Please select the one below that is the best fit for your research. If you are not sure, read the appropriate sections before making your selection.

- [x] Life sciences
- [ ] Behavioural & social sciences
- [ ] Ecological, evolutionary & environmental sciences

For a reference copy of the document with all sections, see nature.com/documents/nr-reporting-summary-flat.pdf

Life sciences study design

All studies must disclose on these points even when the disclosure is negative.

Sample size	For population genomic and GWAS analyses, 305 different spinach accessions were used. These accessions were selected based on their availability in the germplasm collection and are sufficient for population and GWAS analyses based on literature.
Data exclusions	One cultivated (USX) and two wild (PI 647862 and PI 608712) accessions, whose taxonomy could be misclassified, were excluded from the population genomic analyses.
Replication	Two biological replicates were used for transcriptome sequencing, which were used to aid genome annotation. three replicates, each with 10 independent plants were used for phenotyping. All attempts at replication were successful.
Randomization	For phenotyping, spinach accessions were planted in the field using a randomized complete block design with three replicates.
Blinding	Not applicable. For a plant genome and population study, blinding is not necessary

Reporting for specific materials, systems and methods

We require information from authors about some types of materials, experimental systems and methods used in many studies. Here, indicate whether each material, system or method listed is relevant to your study. If you are not sure if a list item applies to your research, read the appropriate section before selecting a response.

Materials & experimental systems

n/a	Involved in the study	
x		Antibodies
x		Eukaryotic cell lines
x		Palaeontology and archaeology
x		Animals and other organisms
x		Human research participants
x		Clinical data
x		Dual use research of concern

Methods

n/a	Involved in the study	
x		ChIP-seq
x		Flow cytometry
x		MRI-based neuroimaging
